# On Polypus of the Œsophagus

**Published:** 1859-02

**Authors:** 


*On Polypus of the Œsophagus.* By Prof. A. H. Middeldorpff, of Breslau.

The interesting monograph of Prof. Middeldorpff on polypus of the œsophagus, contains a complete *resumé* of the state of the science on this subject. We extract from it the following highly interesting case, observed by the author:

Jos. Jaensch, born in 1811, lost, accidentally, when young, the left eye, and a few years later, the left arm. He afterwards enjoyed good health, abstaining from excess of food and drink, and avoiding, particularly, irritating substances. In the spring of 1851 he contracted a violent catarrh, which rendered him deaf for a long time. A catarrhal inflammation of the pharynx remained, and embarrassed deglutition, particularly that of dry bread; excretion of pituitous liquid between the meals; sensations of pressure at the epigastrium and behind the sternum; painless dysphagia, but gradually increasing, very marked in certain positions of the patient, and accompanied by eructation, cough, and dyspnœa; the difficulty of deglutition daily augmented; finally, the ingestion of liquid aliments was alone possible. During an attack of violent cough, the patient once vomited sanguinolent mucus.

At the end of the year 1852, the trouble of deglutition having reached its intensity, he drank water copiously, and was seized with violent vomiting, during which a body resembling a kidney was raised into the mouth between the teeth. An intense dyspnœa followed. The patient succeeded in swallowing the body in question, and applied for medical aid. When Prof. Middeldorpff first saw the patient, he was pale and much emaciated; the finger, introduced into the pharynx to the posterior wall of the larynx, did not come in contact with any foreign body; a probe, of half a finger's diameter, was passed down in the median line, and in directing it to the right side, an obstacle was found, which could be easily overcome, and was not very deeply seated.

On the 14th January, 1853, Prof. Middeldorpff directed the patient to take a large quantity of warm water and an emetic. During the violent vomiting which ensued, a turgid and violaceous body appeared between the dental arches — this was the polypus. Prof. Middeldorpff seized it with the forceps of Museux, drew it toward the left commissure of the mouth, in consequence of which the difficulty of respiration was somewhat diminished; a ligature of waxed silk thread was applied over the tumor, at a level with the base of the tongue. The operation was attended with repeated vomitings and great dyspnœa. The polypus was afterwards divided with the scalpel, three-quarters of an inch above the ligature; it became turgescent, of purple color, and a great quantity of blood escaped from it; in a short time its color sensibly diminished. The patient afterwards swallowed the pedicle of the tumor and the ligature, the extremities of which were attached around the left ear; the vomiting, oppression, and dyspnœa ceased immediately; the patient felt very comfortable; it was ascertained, on repeated trial, that slight traction at the end of the ligature produced pain. The patient was ordered not to touch the thread, to use fluid and cold food, and to present himself every second day. Nothing remarkable occurred up to the eighteenth day after the extirpation. At this time, the loop of the ligature rose into the mouth; it measured twelve millimètres in diameter, and thus encircled a pedicle of about thirty-seven millimètres in circumference. From this moment the troubles of the patient ceased; deglutition and respiration were unembarrassed; the appetite soon revived, and the patient gained in strength and flesh.

Prof. Middeldorpff saw his patient five years after the operation; his health was then excellent; the excretion of pituitous liquid had ceased. On external examination of the neck, nothing abnormal was discoverable; a probe of eight lines diameter still met with a slight obstacle at a level with the larynx; in short, the health of the patient was perfectly established.

*Examination of the Tumor.* The excised tumor measured eight centimètres in length, and four in thickness, and weighed about forty grammes; it was cylindrical; its smooth and shining surface presented some inequalities and excoriations, which bled easily; it was covered externally by a layer of stratified pavement epithelium; underneath this a layer of conical papillæ was detected; the papillæ were visible to the naked eye, and arranged with great regularity in a spiral around the longitudinal axis of the tumor, and received vessels at their base; underneath the papillary layer, the proper substance of the tumor was situated; Prof. Reichert found it, on examination, very vascular, but without nerves; it was composed of connective tissue, in which the cellular elements were still very visible: in other words, a connective tissue, not yet arrived at maturity; here and there fat globules, either free or in cells, were detected; it was, in short, a vascular fibrous tumor, covered with papillæ. By comparing the length of the extirpated portion with that of the remaining portion of the polypus, and by making repeated measurements and explorations in the dead body, Prof. Middeldorpff arrived at the conclusion, that the extremity of the polypus was situated about two inches from the carotid, and that the pedicle was attached at about a level with the larynx. Although these data are merely approximative, they harmonize, in a remarkable manner, with the results furnished by the catheterism.

Prof. Middeldorpff adds, that Dr. John was the first to describe polypi of the naso pharyngeal papillæ, in his dissertation, *De Polypis Narium;* Varsoviæ, 1855.—*De Polypis Œsophagi*, *Breslau, and Gazette Hebdomad.*, *Oct.* 8, 1858, from *The N. A. Medico-Chirurgical Review.*

